# Supplementary material for: Association between prehospital field to emergency department delta shock index and in-hospital mortality in patients with torso and extremity trauma: A multinational, observational study
Source: PLoS One. 2021 Oct 25;16(10):e0258811. doi: 10.1371/journal.pone.0258811 (PMC8544870; doi:10.1371/journal.pone.0258811)
Supplement: S1 Table — (DOCX) [file pone.0258811.s001.docx]

**Supplementary table 1. Association between exposure groups and outcomes in multivariate logistic regression.**

|  | Embolization | |
| --- | --- | --- |
|  | Unadjusted OR (95% CI) | Adjusted OR (95% CI)* |
| DSI ≤0.1 | Reference | Reference |
| DSI >0.1 | 3.14 (1.87-5.25) | 3.15 (1.87-5.30) |
|  | Surgery | |
| DSI ≤0.1 | Reference | Reference |
| DSI >0.1 | 1.22 (1.06-1.40) | 1.29 (1.12-1.49) |

Abbreviations: OR, odds ratio; CI, confidence interval; DSI, delta shock index

^*^Adjusted for age, sex, EMS time, mechanism of injury, intent of injury, and location of injury
