# Supplementary material for: Association between prehospital field to emergency department delta shock index and in-hospital mortality in patients with torso and extremity trauma: A multinational, observational study
Source: PLoS One. 2021 Oct 25;16(10):e0258811. doi: 10.1371/journal.pone.0258811 (PMC8544870; doi:10.1371/journal.pone.0258811)
Supplement: S2 Table — (DOCX) [file pone.0258811.s002.docx]

**Supplementary table 2. Association between exposure groups and outcomes according to prehospital shock index in multivariate logistic regression.**

|  | | Embolization | |
| --- | --- | --- | --- |
|  |  | Unadjusted OR (95% CI) | Adjusted OR (95% CI)* |
| EMS SI <=0.9 | DSI ≤0.1 | Reference | Reference |
|  | DSI >0.1 | 2.79 (1.57-4.98) | 2.90 (1.62-5.19) |
| EMS SI >0.9 | DSI ≤0.1 | 2.21 (0.98-4.96) | 3.00 (1.31-6.86) |
|  | DSI >0.1 | 16.4 (6.33-42.3) | 19.0 (7.17-50.3) |
|  | | Surgery | |
| EMS SI <=0.9 | DSI ≤0.1 | Reference | Reference |
|  | DSI >0.1 | 1.18 (1.02-1.37) | 1.29 (1.11-1.50) |
| EMS SI >0.9 | DSI ≤0.1 | 1.71 (1.44-2.03) | 2.40 (2.00-2.87) |
|  | DSI >0.1 | 3.63 (2.44-5.42) | 4.70 (3.01-7.14) |

Abbreviations: OR, odds ratio; CI, confidence interval; DSI, delta shock index

^*^Adjusted for age, sex, country, EMS time, mechanism of injury, intent of injury, and location of injury
