# Supplementary material for: Association between prehospital field to emergency department delta shock index and in-hospital mortality in patients with torso and extremity trauma: A multinational, observational study
Source: PLoS One. 2021 Oct 25;16(10):e0258811. doi: 10.1371/journal.pone.0258811 (PMC8544870; doi:10.1371/journal.pone.0258811)
Supplement: S1 Appendix — (DOCX) [file pone.0258811.s003.docx]

**S1 Appendix**

PATOS Clinical Research Network

Participating Nation Investigators: T.V. Ramakrishnan (India), Sabariah Faiwah Jamaluddin (Malaysia), Hideharu Tanaka (Japan), Bernadett Velasco (Philippines), Ki Jeong Hong^4,5^ (South Korea), Jen Tang Sun (Taiwan), Pairoj Khruekarnchana (Thailand), Saleh Fares LLC (United Arab Emirates), Do Ngoc Son (Vietnam)

Participating Site Investigators: Ramana Rao (GVK EMRI, India), George P. Abraham (Indian Institute of Emergency Medical Services, India), T.V. Ramakrishnan (Sri Ramachandra Medical Center, India), Sabariah Faiwah Jamaluddin (Sungai Buloh Hospital, Malaysia), Mohd Amin Bin Mohidin (Sultanah Aminah Hospital, Malaysia), Al-Hilmi Saim (Seri Manjung Hospital, Malaysia), Lim Chee Kean (Pulau Pinang Hospital, Malaysia), Cecilia Anthonysamy (Serdang Hospital, Malaysia), Shah Jahan Din Mohd Yssof (Kuala Lumpur Hospital, Malaysia), Kang Wen Ji (Sarikei Hospital, Malaysia), Cheah Phee Kheng (Sabah Women and Childrens Hospital, Malaysia), Shamila bt Mohamad Ali (Ampang Hospital, Malaysia), Periyanayaki Ramanathan (Kajang Hospital, Malaysia), Chia Boon Yang (Miri Hospital, Malaysia), Hon Woei Chia (Sarawak General Hospital, Malaysia), Hafidahwati binti Hamad (Queen Elizabeth II Hospital, Malaysia), Samsu Ambia Ismail (Teluk Intan Hospital, Malaysia), Wan Rasydan B. Wan Abdullah (Raja Perempuan Zainab II Hospital, Malaysia), Hideharu Tanaka (Kokushikan University, Japan), Akio Kimura (National Center for Global Health and Medicine Hospital, Japan), Bernadett Velasco (East Avenue Medical Center, Philippines), Carlos D. Gundran (Philippine College of Emergency Medicine, Philippines), Pauline Convocar (Southern Philippines Medical Centre, Philippines), Nerissa G.Sabarre (Pasig City General Hospital, Philippines), Patrick Joseph Tiglao (Corazon Locsin Montelibano Memorial Regional Hospital, Philippines), Ki Jeong Hong^4,5^ (Seoul National Univerisity Hospital, South Korea), Kyoung Jun Song^3,5^ (Boramae Medical Center, South Korea), Joo Jeong^1,5*^ (Seoul National Univerisity Bundang Hospital, South Korea), Sung Woo Moon and Joo-yeong Kim (Korea University Ansan Hospital, South Korea), Won Chul Cha (Samsung Medical Center, South Korea), Seung Chul Lee (Dongguk University Ilsan Hospital, South Korea), Jae Yun Ahn (Kyungpook National University Hospital, South Korea), Kang Hyeon Lee (Wonju Severance Christian Hospital, South Korea), Seok Ran Yeom (Pusan National University Hospital, South Korea), Hyeon Ho Ryu (Chonnam National University Hospital, South Korea), Su Jin Kim (Korea University Anam Hospital, South Korea), Sang Chul Kim (Chungbuk National University Hospital, South Korea), Lin-Chen Chiang (National Taiwan University Hospital, Taiwan), Jen Tang Sun (Far Eastern Memorial Hospital, Taiwan), Ruei-Fang Wang (Shin Kong Wu Ho-Su Memorial Hospital, Taiwan), Shang-Lin Hsieh (Mackay Memorial Hospital, Taiwan), Wei-Fong Kao (Taipei City Hospital, Taiwan), Sattha Riyapan (Faculty of Medicine Siriraj Hospital, Thailand), Parinya Tianwibool (Faculty of Medicine Chiangmai University, Thailand), Phudit Buaprasert (Faculty of medicine Vajira hospital, Navamindradhiraj University, Thailand), Osaree Akaraborworn (Prince of Songkla University, Thailand), Omer Ahmed Al Sakaf (Dubai Coorporation for Ambulance Services, United Arab Emirates), Saleh Fares LLC (National Ambulance, United Arab Emirates), Le Bao Huy (Thong Nhat Hospital, Vietnam), Do Ngoc Son (Bach Mai Hospital, Vietnam), Nguyen Van Dai (Viet Tiep Hospital, Vietnam)

^1^ Department of Emergency Medicine, Seoul National University Bundang Hospital, South Korea

^2^ Department of Emergency Medicine, Seoul National University College of Medicine, South Korea

^3^ Department of Emergency Medicine, Seoul National University Boramae Medical Center, South Korea

^4^ Department of Emergency Medicine, Seoul National University Hospital, South Korea

^5^ Laboratory of Emergency Medical Services, Seoul National University Hospital Biomedical Research Institute, South Korea

^6^ Department of Emergency Medicine, Universiti Teknologi MARA Sungai Buloh Campus, Malaysia

* Corresponding author:

E-mail: joojeong@snubh.org
